# Two Different Extranodal Lymphomas in an HIV^+^ Patient: A Case Report and Review of the Literature

**DOI:** 10.1155/2019/8959145

**Published:** 2019-09-26

**Authors:** Clara Bertuzzi, Elena Sabattini, Francesco Bacci, Claudio Agostinelli, Gian Gaetano Ferri

**Affiliations:** ^1^Hematopathology Unit, Sant'Orsola University Hospital, Bologna, Italy; ^2^ENT and Audiology Unit, Department of Experimental, Diagnostic and Specialty Medicine, Sant' Orsola Hospital, University of Bologna, Bologna, Italy

## Abstract

Human immune deficiency virus- (HIV-) infected individuals present a higher risk of developing malignancies. Herein, we are presenting an unusual case of an untreated HIV^+^ patient, who developed two distinct lymphoproliferative disorders in a period of 4 years: a primary cutaneous T-cell lymphoma (PCTCL) and a diffuse large B-cell lymphoma (DLBCL) not otherwise specified (NOS), the latter developed while commencing combined antiretroviral therapy (cART). The two lymphomas also showed peculiar features: PCTCL are rarely described in HIV^+^ setting and particularly at such a low clinical stage, and the DLBCL showed uncommon cytology, non-GCB phenotype, EBER negativity, and absence of c-MYC translocation, all atypical features in this clinical context. This report not only confirms the increased risk of lymphoma for HIV^+^ patients and HIV infection being one of the major risk factors for lymphoid disorders but draws the attention on the possible occurrence of unusual features, suggesting that HIV serology should always be investigated in the clinical suspicion of lymphoma.

## 1. Introduction

Human immune deficiency virus- (HIV-) infected individuals present a higher risk of developing malignancies. Among these latter, lymphomas are the most frequent neoplasms in USA and Europe within seropositive patients [1, 2]. Furthermore, aggressive B-cell lymphomas together with Kaposi's sarcoma and invasive cervical cancer are considered to be acquired immune deficiency syndrome (AIDS)-defining cancers [3]. Here, we are presenting an unusual case of an HIV^+^ patient who developed two separate extranodal lymphomas and reviewing the literature focusing on lymphoproliferative disorders in the setting of HIV infection.

## 2. Case Presentation

**Figure 1:**
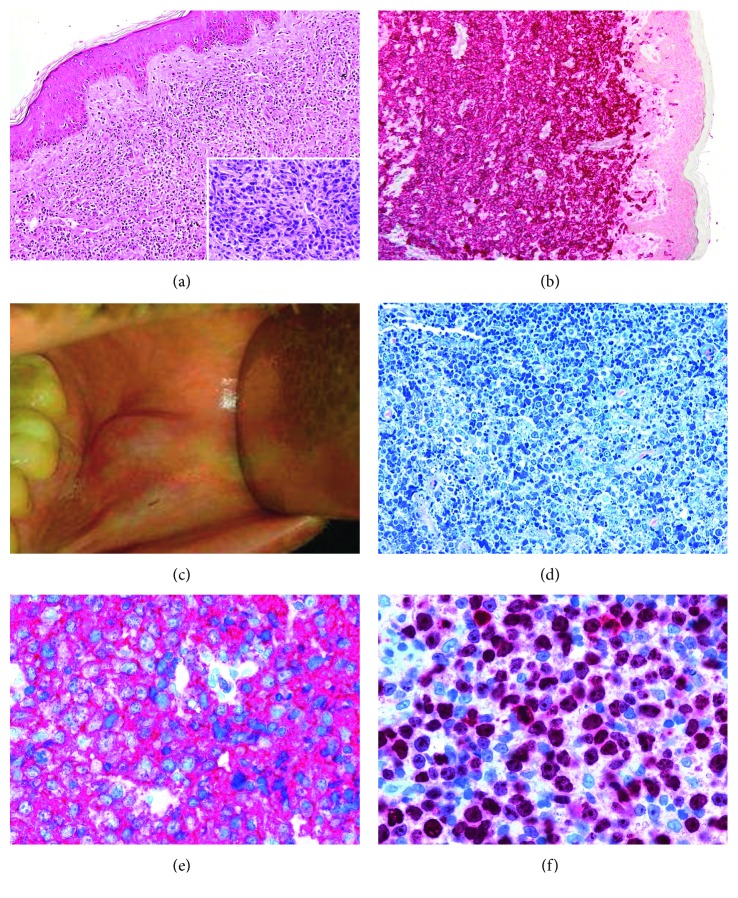
(a) Morphological features (haematoxylin and eosin ×100; insert ×400) and (b) CD30 expression (×100) of the skin lesion; (c) intraoperatory picture of gingival neoformation and (d) its morphologic features (Giemsa stain, ×200); immunohistochemical expression of (e) CD20 (×400) and (f) Ki67 (×400) in DLBCL.

In March 2010, a 43-year-old man underwent surgery to remove a subcutaneous nodule from the inguinal region. The patient had a known hepatitis B virus (HBV) and HIV infection since 1986; he had never been treated with a combined antiretroviral therapy (cART) and had never developed opportunistic infections. The histological examination of the removed lesion showed a proliferation of medium/large sized lymphoid cells infiltrating dermis and subcutaneous tissue, without epidermotropism or ulceration and absence of inflammatory background (Figure 1(a)). Immunohistochemical investigations of these cells showed a T-lymphoid phenotype: CD3^+^, CD2^+^ with partial expression of CD5, CD7, and TIA1. CD4, CD8, and granzyme B were negative. The tumoral population was highly proliferating (90% Ki67^+^ cells). A strong and diffuse CD30 positivity was observed (Figure 1(b)), while B-cell markers were negative as well as EMA, Perforin, Alkc, HHV8, and CD56. Staging procedures failed to demonstrate other sites of involvement. The uniqueness and persistence of the lesion, the absence of ulceration, and the strong diffuse positivity for CD30 prompted a diagnosis of primary cutaneous CD30^+^ T-cell lymphoproliferative disorders with the features of anaplastic large cell lymphoma. A targeted radiotherapy treatment was suggested.

In November 2013, the patient started cART and almost simultaneously he developed a hard, nonpainful, nonulcerated gingival lesion sited on the left anteroinferior genus mucosa. Few months later, this lesion had reached 3 cm in diameter (Figure 1(c)) and an incisional surgical biopsy was performed (February 2014). The gingival tissue was diffusely infiltrated by large lymphoid cells with a prevalent immunoblastic morphology (Figure 1(d)), expressing CD20 (Figure 1(e)), BCL6, and IRF4 while CD10, CD3, and CD30 were negative. The proliferation index was high (90% Ki67^+^ cells) (Figure 1(f)). BCL2 resulted diffusely positive while cMYC protein was detected in 25% of the 57 cells and was thus considered negative, being the established cutoff positivity ≥40%. In situ hybridization analysis for Epstein–Barr virus (EBV) small encoded RNAs did not reveal EBV presence. The investigation for cMYC rearrangements by fluorescence in in situ hybridization was negative. Based on the molecular and morphophenotypic findings, a diagnosis of diffuse large B-cell lymphoma (DLBCL) not otherwise specified (NOS) with non-GCB phenotype was made, according to Hans' algorithm [4]. The staging positron emission tomography showed areas of hypercaptation in the left pleura and in the right obturatory site, indicating a multifocal disease. The patient refused the proposed treatment and was lost at follow-up.

## 3. Discussion

Lymphomas are one of the most common cancers among patients with HIV/AIDS [5, 6]. Before the introduction in 1996 of cART, the estimated risk to develop a non-Hodgkin lymphoma was 60–200 times higher in the HIV^+^ individuals than in the general population [7]. Since the advent of cART, the mortality caused by infections has decreased and HIV-patients' life expectancy is now close to normal. However, despite the overall reduced incidence of lymphomas, these neoplasms are still the most important cause of cancer-related death, and HIV infection is so far the strongest risk factor for the onset of non-Hodgkin B-cell lymphoma (B-NHL) [5, 6].

The most frequent HIV-associated lymphomas are DLBCL (often involving the primary central nervous system) and Burkitt lymphoma (BL), both AIDS-defining illnesses, and accounts for 50% and 40% of NHLs, respectively [8, 9]; among non-AIDS-defining malignancies, Hodgkin's lymphoma (HL) is one of the most common [8]. The World Health Organization has grouped the HIV-associated lymphomas into three categories: [8] (a) lymphomas also occurring in immunocompetent patients such as BL, DLBCL, HL, plasmablastic lymphoma (PL), and other rarer subtypes (mycosis fungoides, anaplastic large cell lymphoma, NK/T-cell lymphoma, marginal zone lymphoma, and lymphoplasmacytic and lymphoblastic lymphoma); (b) lymphomas occurring more specifically in HIV-infected patients such as primary effusion lymphoma (PEL), PL, and human herpes virus-8 (HHV8) positive DLBCL; (c) lymphomas occurring in other immunodeficiency states, in which 84 include lymphoid proliferations resembling posttransplant lymphoproliferative disorders. Interestingly, the latter are far less common in the HIV setting than in transplanted patients, where they represent less than 5% of lymphomas [8].

The high prevalence of NHL also in cART era hints that additional mechanisms are involved in HIV lymphomagenesis beyond impaired immune surveillance. The chronic B-cell activation favoured by the HIV-mediated immune dysfunction, which is responsible for hypergammaglobulinemia, defective humoral immunity, and florid germinal centre hyperplasia (all characterizing early HIV-infection), was demonstrated to be critical for the development of lymphomas. This persistent polyclonal proliferation of B cells increases the risk of mistakes involving oncogenic genetic alterations during the germinal centre reaction. Chronic B-cell stimulation might be linked to: (a) HIV itself and various HIV-encoded proteins, (b) the Tat-induced abnormal production of interleukin 6 (IL-6) and IL-10, (c) the antigenic stimulation of opportunistic viral infections (i.e., Epstein–Barr virus (EBV), HHV8), and (d) the incorporation of host-derived CD40 L into virions and the ability of these latter to bind B lymphocytes mimicking the endogenous CD40-CD40 L interaction [10–15]. Furthermore, HIV preferentially infects T-follicular helper (TFH) cells altering B-cell development and function and the control of chronic viral infection [16, 17]. It was recently shown that expanded TFH-cell populations in HIV^+^ patients are associated with an increase in germinal centre B-cells and plasma cells and a decrease of memory B-cells and immunoglobulin hypersecretion [18, 19]. HIV may also act as a local lymphomagenic factor through the effects of the virus encoded and secreted proteins. Tat functionally interacts with and enters into B lymphocytes, leading to deregulation of the pRb2/p130 oncosuppressor protein [20]. Variants of the HIV-1 matrix protein p17 (vp17s) recently detected in the NHL samples of HIV^+^ patients, besides its impact on angiogenesis, displays also B-cell growth-promoting activity, due to activation of the Akt signaling pathway, suggesting its possible role in oncogenic transformation [21]. These vp17s are more frequently detected in HIV^+^ patients with NHL than in patients without NHL. Moreover mice transgenic overexpressing the HIV proteins p17, gp120, and nef, develop B-cell lymphomas [22], and induction of RAG1 gene expression in costimulated human B-cells, treated with p17, has been recently observed. In this specific setting of costimulation, the activation of RAG genes could lead to genomic instability and transformation [23].

We report an unusual case of an HIV^+^ patient who developed two distinct lymphoproliferative disorders in a period of 4 years after 25 years of HIV untreated and uncomplicated infection: a primary cutaneous T-cell lymphoma disorder and, three years later, an oral DLBCL, at the time he started cART.

In the HIV^+^ population, DLBCL presents more frequently with disseminated disease (stages III and IV) and extranodal presentation than in HIV^−^ people [8], the most common sites being the gastrointestinal tract, liver, bone marrow, and central nervous system (CNS). In particular, the risk of developing a primary CNS lymphoma (PCNSL) was 1000 times higher in the HIV^+^ patients than in controls before cART although PCNSL still accounts for 20% of HIV-associated lymphomas and generally occurs in patients with very low CD4 cell counts (<100 cells/*µ*l). HIV-related DLBCL usually consists of large cells characterized by centroblastic or immunoblastic cytology and displays a complete B-cell phenotype (CD20^+^/CD19^+^/CD79a^+^/PAX5^+^) [8], with a more frequent germinal centre B-cell (GCB) phenotype, although the prognostic impact of the cell of origin is not clear in the HIV setting [8]. Up to 80% of the cases are EBV^+^, and this association reaches 100% in PCNSL [8]. The EBV-infected blasts carry a latency type II with expression of latent membrane protein 1 (LMP1). LMP1 and the mutation/deletion of TNFAIP3 gene, commonly observed in HIV^+^ DLBCL, may contribute to lymphoma genesis activating the nuclear factor kB (NF-kB) pathway and consequently promoting cell proliferation and survival [8]. Additionally, cMYC and BCL6 gene rearrangements are frequently detected as well as aberrant somatic hypermutations involving PAX5, Rho/TTF, and PIM1 genes [8].

Our patient developed an extranodal DLBCL at the beginning of cART, when CD4 count was 437 cells/*µ*l, and the PET scan revealed a disseminated disease. The finding of a rapidly growing neoformation in the oral cavity of an HIV^+^ individual generally suggests an EBV^+^ PL, which is the most common lymphoma arising at this site and setting. However, one should keep in mind that LBCL other than EBV + PL localizes in oral cavity, particularly in HIV-infected subjects, and therefore any oral LBCL should per se prompt clinical investigations for HIV infection. The described DLBCL showed peculiar features: in fact, it presented with immunoblastic (IB) cytology and a non-GCB phenotype, which are biologic features commonly associated to EBV positivity, which was however negative in our case by means of in situ hybridization analysis for EBV small encoded RNAs, although Mundo and colleagues [24] reported possible false negative results. We did not perform RNA scope or miRNAs qPCR and can not thus exclude the possibility of a previous EBV infection followed by the virus loss (so called “hit and run” event). Nonetheless, we considered our observations consistent, also considering the patient's CD4+ lymphocyte blood count which was still relatively high for an untreated long-lasting HIV infection, which had never been complicated by opportunistic infections. The cMYC rearrangement that is reported in about 20% of HIV-related IB-DLBCL was not revealed by FISH analysis in our case.

Interestingly, a primary cutaneous peripheral T-cell lymphoma (PC-PTCL) was diagnosed three years before the onset of gingival DLBCL. Although relative risk of developing NHL is increased among HIV^+^ population, PTCL are rarely described; most data referred to small retrospective series and suggest a significantly lower incidence than the B-cell ones [25–27]. The PTCL frequency is estimated between 2% and 7% of all HIV-associated lymphoma, and it seems to be similar in pre- and post-cART era [25–27], with not otherwise specified and ALK-anaplastic large cell lymphoma types being mostly prevalent, and angioimmunoblastic T-cell lymphoma (AITL) sporadically found. Patients affected by systemic PTCL usually have advanced stage disease, multiple extranodal involvement, B-symptoms, poor performance status, and a haemophagocytic syndrome sometimes complicating the clinical course. [25–28]. Cutaneous T-cell lymphomas rarely occurs in HIV-infected patients: mycosis fungoides (MF) is definitely unusual, whereas primary cutaneous CD30^+^ T-cell lymphoma ALCL type (PC-ALCL) seems to be more frequent [29–31]. The latter more frequently arises in severely immunocompromised patients with very low CD4 count and presents with a single deep skin, rapidly growing nodule. These HIV^+^ cases share the same morphologic and phenotypic features as in HIV^−^ patients, although they more often express T-cell antigens (i.e., CD2, CD3, and CD4) [28–30]. Occasionally EBV positivity was reported, while HHV8 is regularly negative [30, 31]. Isolated lesions are treated with surgical excision or radiation therapy or both, while multifocal skin lesions may require multiagent chemotherapy [29–31]. Survival was correlated to the immune-suppression status and is good in patients without opportunistic infections. Occasional spontaneous and/or complete regression after immune recovery secondary to cART was also reported. In the presented case, the skin lesion consisted of large atypical cells diffusely and intensively expressing CD30. The T-cell origin was demonstrated by the expression of CD2, CD3, CD5, and CD7; however, the neoplasm turned out to be negative for both CD4 and CD8. This phenotypic profile suggested a diagnosis of CD30 + PTCL, which might be referred to as ALCL, despite negativity for EMA and cytotoxic markers. The primary cutaneous origin was ascertained by the clinical staging and the bone marrow biopsy which excluded any systemic involvement, and a targeted radiotherapy was suggested.

In conclusion, we presented an extremely unusual case of an HIV^+^ patient who developed both a B-cell and T-cell neoplasms in a relatively short period of time, with one of them (PC-ALCL) being an unusual variant in the setting of HIV infection especially with still good clinical conditions and relatively high CD4 count. This case provides confirmation of higher exposure of HIV^+^ patients to the lymphoma development, sometimes presenting with unusual biologic features, and of HIV being by itself one of the major risk factors for lymphoid disorders, indicating that the HIV status should always be investigated in the clinical suspicion of lymphoma.
